# The contribution of muscle spindles to position sense measured with three different methods

**DOI:** 10.1007/s00221-023-06689-4

**Published:** 2023-08-31

**Authors:** Christopher Roach, Christopher Love, Trevor Allen, Uwe Proske

**Affiliations:** 1 Department of Physiology, Monash University, Clayton, Victoria 3800 Australia; 2 Accident Research Centre, Monash University, Clayton, Victoria 3800 Australia

**Keywords:** Muscle spindle, Thixotropy, Proprioception, Self-awareness

## Abstract

The sense of limb position is important, because it is believed to contribute to our sense of self-awareness. Muscle spindles, including both primary and secondary endings of spindles, are thought to be the principal position sensors. Passive spindles possess a property called thixotropy which allows their sensitivity to be manipulated. Here, thixotropic patterns of position errors have been studied with three commonly used methods of measurement of position sense. The patterns of errors have been used as indicators of the influence exerted by muscle spindles on a measured value of position sense. In two-arm matching, the blindfolded participant indicates the location of one arm by placement of the other. In one-arm pointing, the participant points to the perceived position of their other, hidden arm. In repositioning, one of the blindfolded participant’s arms is placed at a chosen angle and they are asked to remember its position and then, after a delay, reproduce the position. The three methods were studied over the full range of elbow angles between 5° (elbow extension) and 125° (elbow flexion). Different outcomes were achieved with each method; in two-arm matching, position errors were symmetrical about zero and thixotropic influences were large, while in one-arm pointing, errors were biased towards extension. In repositioning, thixotropic effects were small. We conclude that each of the methods of measuring position sense comprises different mixes of peripheral and central influences. This will have to be taken into consideration by the clinician diagnosing disturbances in position sense.

## Introduction

Human position sense is one of a group of senses, called the proprioceptive senses, which are generated by our own actions during everyday behaviour. Traditionally, included under the heading of proprioceptive senses are the senses of limb position and movement, collectively referred to as the kinaesthetic senses, the senses of muscle force, heaviness, effort and balance (Proske and Gandevia 2012). Arguably, the most important of the proprioceptive senses is the sense of position. It allows us to know where our limbs and bodies are in relation to each other and to their surroundings, without needing to look at them, giving us the ability to safely navigate obstacles and, if necessary, take evasive action. It has been speculated (Cole 1995, 2016) that the sense of position is a fundamental aspect of our self-awareness, the “sense of self”. The proprioceptive senses, including the sense of position, are not easy to study, because they operate largely at the unconscious level and to bring them out it is necessary to specifically interrogate each sense, individually.

We have chosen here to study, specifically, position sense, because of its importance in everyday life. We have not considered the equally important movement sense. Looking at the various methods employed in the many published studies of position sense, we have identified three categories of methods. Of course, within each category, there are often considerable differences in approach taken by experimenters; we have focussed on features frequently present with a particular method to determine its classification. Our conclusions about such classifications are not necessarily agreed upon by everyone and others have come to rather different conclusions (see, for example, Han et al. 2016).

**Fig. 1:**
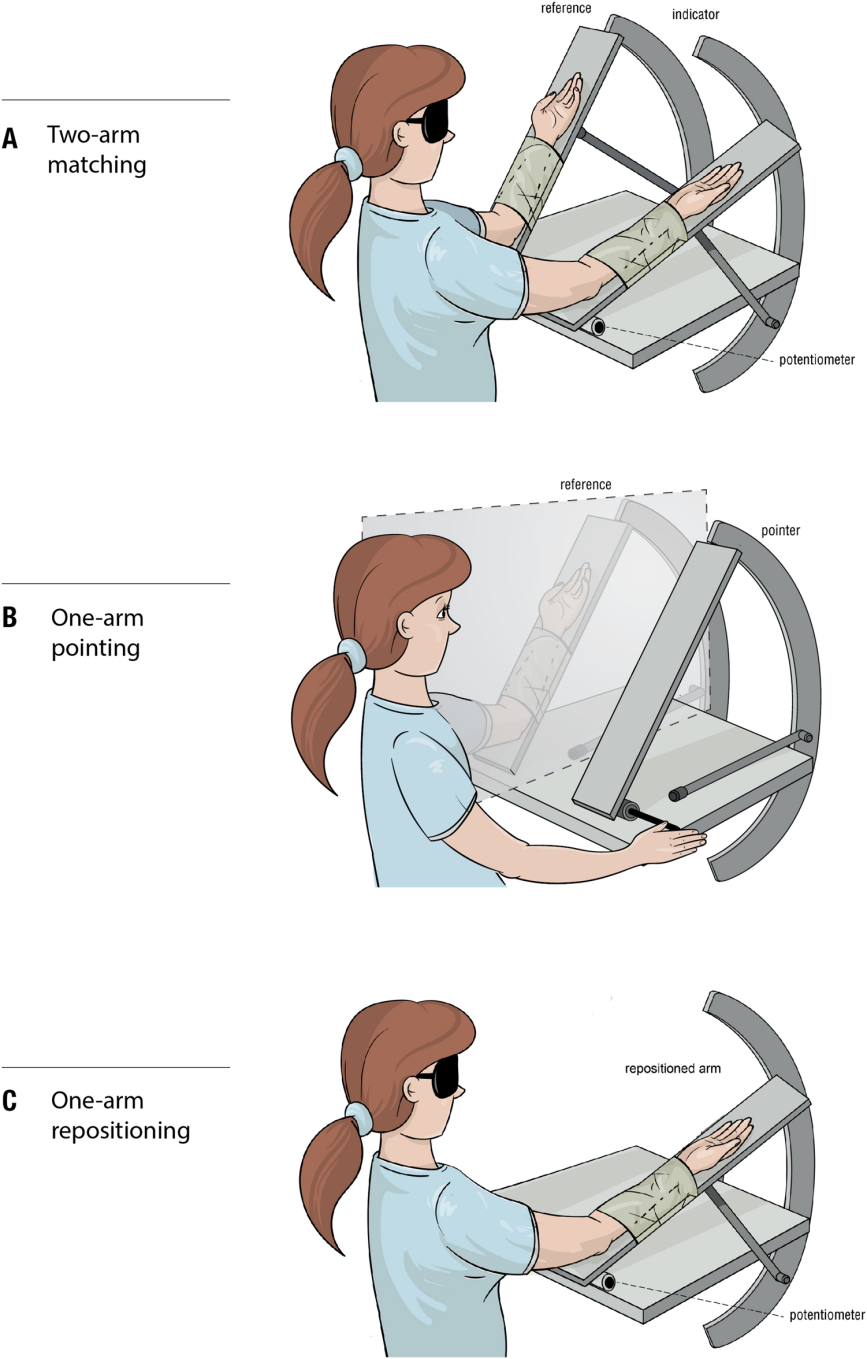
The apparatus. **A** Matching. The blindfolded subject was seated, with both forearms strapped to paddles by means of Velcro straps attached at the wrist and forearm. The paddles could be moved in the vertical (sagittal) plane about hinges fixed to a baseplate. Potentiometers at the hinges provided a continuous output signal of elbow angle. Both paddles could be locked in position at a chosen angle by means of metal struts that could be fixed with locking pins to a curved supporting scaffold, bolted to the back of the apparatus. Contraction force was measured with transducers attached to the struts. At the start of each matching trial the subject had to carry out conditioning isometric contractions of forearm muscles of both arms. For this the paddles had to be locked in position. The arms were conditioned by asking the subject to carry out a half-maximal, isometric contraction, of elbow flexors and extensors, 2 s in duration, at 125° or 5°. If the reference arm was conditioned at 125°, the indicator was conditioned at 5°, or vice versa. After arm muscles had relaxed, the reference arm was unlocked and moved to a chosen test angle. At the same time, the indicator was unlocked and the subject was asked to hold their reference arm steady while its position was matched by placement of the indicator. **B** Pointing. Here, the reference arm was hidden behind a screen. The subject wore an eye patch over the eye closest to the reference arm so they were unable to see any part of their hidden arm or shoulder. The apparatus used two paddles, as before, but only the reference arm was strapped to a paddle by means of Velcro straps at the wrist and forearm. As before, the subject was asked to carry out conditioning isometric contractions of reference arm muscles at 125° or 5°. After arm muscles had relaxed, the arm was unlocked and moved to a chosen test angle. The subject held the reference arm in position while, with their other arm, they moved the second paddle by rotating a shaft at its base. When they felt that the pointer paddle was aligned with the position of the hidden forearm, they declared it and the experimenter marked the point on the angle trace on the computer screen. Re-drawn from Chen et al. (2021). **C** Repositioning. For this experiment, only a single paddle was required. The chosen reference arm was strapped to the paddle and, in some trials, it was conditioned with a co-contraction of elbow muscles at 125° or 5°. In other trials the arm was left unconditioned. The experimenter moved the blindfolded subject’s arm to a chosen test angle, in the direction of extension when the start angle was 125° and into flexion when the start angle was 5°. The arm was left at the test angle for 2 s, which the subject was asked to remember, before it was brought it back to its starting point. Two seconds later the subject had to reposition the arm at the remembered test angle. The arm remained unconditioned before the remembering and repositioning stages (‘none’), or it was conditioned with a half-maximum voluntary contraction after the remembering stage, (‘after’), or both before and after the remembering stage (‘before and after’)

The method first introduced to measure position sense at the forearm was the two-arm matching task. One arm is placed at a chosen test angle and the blindfolded participant is asked to match its position by placement of the other arm. The method is historically important, because it provided the first evidence for muscle spindles as position sensors (Goodwin et al. 1972). The second method is one-arm pointing. The participant uses a pointer with one arm to indicate the perceived position of the other arm, hidden behind a screen (Fig. 1). The third method is that of repositioning arm position (Goble 2010). It is important, because the vast majority of studies, especially those concerned with clinical aspects, make use of it (Horvath et al. 2023). Here, we have tested the simplest version of the method; the blindfolded participant has one arm moved to a chosen test angle by the experimenter and they are asked to remember its position. The arm is then returned to its starting position and, after a delay, the participant is asked to replace it at the remembered location. This is, therefore, a method largely dependent on memory and, in that sense, distinct from two-arm matching or one-arm pointing.

The three methods do not necessarily all measure the same thing. In two-arm matching, it is the position of one-arm relative to the other that is indicated. In one-arm pointing, the subject focusses their attention on the position signal coming from one, unseen arm and indicates its position with a pointer. A repositioning task relies, at least in part, on memory of perceived limb position and the question arises, does this involve the signals of muscle spindles at all?

Over the years, we have introduced a method, thixotropic conditioning, with which we are able to manipulate measured values of position sense (see Proske et al. 2014; Proske and Gandevia 2018 for reviews). The method allows generation of specific, predictable changes in spindle afferent discharges that are expressed in the distribution of position errors (Gregory et al. 1988). Other peripheral sensory receptors do not exhibit thixotropy. In the present study, when using a particular method of measurement of position sense, we have looked for the degree of preservation of a thixotropic pattern in the position errors obtained with that method as evidence for spindles as the source of the position signal.

After a shortening movement of a relaxed muscle, the intrafusal fibres of spindles have been shown to fall slack (Gregory et al. 1988). This acts to lower the resting discharge rate maintained by spindles and, therefore, alters indicated limb position. In order to generate large thixotropic errors at the elbow joint, we have devised a method which minimises the amount of slack present in intrafusal fibres of spindles of one group of elbow muscles and increases it in spindles of the antagonists. It means that after this form of conditioning, spindles of the antagonists are left maintaining very different discharge rates, leading to a large signal difference between them which, in turn, leads to large position errors (Allen et al. 2007).

The degree to which these errors were preserved in the values of position sense, measured with each of the three methods, was used as evidence for persistence of peripheral influences in the measured outcome. That is, the size of the thixotropic error distribution provided some indication of whether or not the measured position signal had undergone significant central processing. Such information is potentially useful in the clinic, since measurements of position sense are used routinely by the neurologist, as one of a battery of tests, when they are trying to decide whether they are dealing with peripheral or central sources of the pathology.

In the present study, we have chosen to make measurements, with each of the three methods, over the full range of angles at the elbow, representing 120° of movement of the forearm. One reason for making measurements over such a wide range is that in earlier experiments of this kind, it was found that the distribution of position errors was somewhat dependent on the angle range over which the measurements had been made (Chen et al. 2021). Revealing new sources of errors in the distribution of position errors at different muscle lengths may help to better understand the mechanisms underlying the generation of this sense.

Our working hypothesis was that with the thixotropic method as used here, position errors in a two-arm matching task would be large; they would be smaller in a one-arm pointing task. In the repositioning task, we predicted that there would be no detectable thixotropic errors, since the position signal was dominated by a memory mechanism that, we presumed, was not susceptible to thixotropy.

## Methods

A total of 22 adult participants enrolled in the study. The experiments were divided into two series since subjects’ concentration levels did not permit acquiring data for all three methods in the one session. There was no evidence of any differences in position sense accuracy between the two groups. In one session, position sense was measured in a two-arm matching and a one-arm pointing task (11 participants), while in the other position, sense was measured in a repositioning task (11 participants). Four of the participants who had carried out both the matching and pointing experiments also took part in the repositioning study.

Six of the 11 participants who participated in the matching and pointing experiments were female. The mean age range of the group was 29.6 (± 15.3) years. Six of the 11 participants who did the repositioning task were female. The mean age of this second group was 21.3 (± 1.3) years. During the participant selection process, anyone with current or past arm injuries and anyone who was unable to maintain concentration throughout the experiment was excluded from the study. In order to take into account any differences in proprioception between the two arms (Goble et al. 2006), in each series, six participants used their dominant arm as the reference, five their non-dominant arm.

Before enrolling in the study, participants gave their written, informed consent. The project received approval of the Monash University Human Ethics Committee (Project ID: 18826) and the ethical aspects of the project conformed with the Declaration of Helsinki.

### Position sense measured by two-arm matching

#### Apparatus

The apparatus used to carry out the matching trials is shown in Fig. 1A. The participant was blindfolded and both forearms were strapped to their respective paddles. The paddles were fixed to the supporting base by means of hinges, which allowed the arms to move vertically, in the sagittal plane, through the full range of elbow angles, 0°–130°, where 0° represented the fully extended arm and 130° the fully flexed arm. Co-linear with each paddle hinge were potentiometers that provided continuous output of elbow angles over the working range, with an accuracy of ± 0.5°. The paddles could be fixed in position at a chosen angle on the supporting scaffolding by means of struts which had locking pins at their ends that could be slid into holes in the scaffolding, over the working range of 5°–125°. Potentiometer output was displayed on a computer screen using Chart software (AD Instruments, Castle Hill, NSW, Australia). Force transducers attached to the struts allowed the experimenter to monitor the strength of the isometric contractions used for muscle conditioning.

#### Two-arm matching task

At the start of a trial, both of the participant’s arms were strapped to the paddles, located so that the elbow joints were in line with the paddle hinges. The participant rested their elbows on cushioned pads to support the weight of their upper arm. The two paddles were locked in position at the conditioning angles, 5° for one arm and 125° for the other. The participant was then asked to carry out voluntary isometric contractions of elbow flexors and extensors of both arms. Here, for each arm, the experimenter asked the participant to exert a 2-s, approximately half-maximum effort contraction in the direction of forearm flexion followed by a similar contraction in the direction of extension. Conditioning of the two arms was carried out simultaneously, with one arm in a fully flexed position, the other in a fully extended position. Conditioning was done simultaneously to minimise possible adaptive changes in the position signal of the reference arm, while it was waiting for the indicator to make a match (Tsay et al. 2014).

After the conditioning contractions, the experimenter unlocked the reference arm and moved it to one of five chosen test angles, 5°, 35°, 65°, 95°, or 125°. The participant was asked to hold the reference arm steady at the test position while the locking pin was removed from the indicator arm. The participant then moved their indicator to align it with the perceived position of the reference arm. They were asked to keep the reference arm stationary throughout this time. Participants inevitably moved their reference slightly before completing the match.

At a given test angle, when the participant had stated that they had made a satisfactory match, the experimenter made a digital mark on the computer trace. The arms were then taken back to the conditioning locations for the next trial. Presentation of test angles was randomised for every participant, and they were tested three times at each angle.

After a series of measurements with one arm conditioned at 5° and the other at 125°, the order of conditioning was reversed, the arm that had been conditioned at 5° was now conditioned at 125° and vice versa for the other arm. Then a second series of measurements at the different test angles was carried out.

Since the arms were conditioned at opposite ends of the angle range, during matching, they always moved towards the matching position from opposite directions. For example, when the reference moved in the direction of extension, its flexors were stretched by the movement; the indicator moved in the opposite direction, in the process stretching its extensors. It meant that the pattern of afferent activity coming from the two arms during the match was very different, one arm with its flexor signal dominating, the other with its extensor signal dominating. That was the intention, to maximise signal differences between the arms, to produce large, thixotropy-dependent errors in position sense.

The conditioning procedure was similar for the three mid-range angles, 35°, 65° and 95°. The reference arm was conditioned at 125° and the indicator at 5°, or vice versa, making for two matching trials at each angle. Conditioning was slightly different for the two extreme angles. When the test angle was 125°, the reference arm was co-conditioned at that angle and was left there. The other arm was also conditioned at 125 and the participant matched position of the reference. Alternatively, the indicator, conditioned at 5°, was moved into flexion to make the match. Therefore, here, the match was made with both arms virtually stationary, or with one-arm stationary while the other moved through the full range of angles to make a match. The same thing was done with the 5° test angle, except here the indicator remained at 5° or moved into extension from 125°.There were, therefore, four trials at the two extreme angles and six trials at the three intermediate angles, making for a total of ten trials. For each angle, the measurements were repeated 3 times, giving a total of 30 trials for each participant.

In order to obtain an indication of the sizes of the position errors directly attributable to thixotropy in two-arm matching, using opposite end conditioning, a series of control measurements was carried out for each participant. For the controls, both arms were conditioned at the same conditioning angle (5° or 125°) and both were moved in the same direction to the same test angle. It meant that the muscles of both arms were in a near-identical thixotropic state. It was, therefore, possible to use these control errors to compare with the errors from opposite conditioning.

#### One-arm pointing task

The equipment used in the pointing trials is shown in Fig. 1B. Here, the reference arm was hidden behind a screen and the participant used their other hand to rotate a lever that moved the pointer paddle. The participant was asked to move the paddle to a position that they felt corresponded to the position of the hidden reference arm. In the pointing experiment the participant wore an eye patch over the eye closest to the hidden arm. It meant that during pointing neither the reference arm nor its shoulder was visible to the participant. Before the reference arm was moved to the test angle, it was co-conditioned at either 5° or 125° and then moved to a chosen test angle, 5°, 35°, 65° 95° or 125° where its position was indicated with the pointer paddle. Therefore, for each test angle, two measurements were made, making for ten measurements for the five angles. A measurement was repeated 3 times so that each participant had to carry out 30 trials.

For the two extreme angles, the participant had to point to an arm co-contracted at 125° and then point to it again when the 125° position had been achieved by movement from 5°. Therefore, at each of the two extreme angles, there were two pointed values, one after conditioning arm muscles at the test angle, the other after conditioning at the opposite end of the angle range and moving to the test angle. It meant that for one of the two measurements of the position signal at each end of the angle range there had been no preceding movement.

For the pointing task, unlike in the matching trials, it was not possible to carry out controls for the effects of the conditioning. The simple control of having no conditioning at all, “none”, as was used in the repositioning trials, was considered unacceptable, since from the start of the experiment the arm would have been in an undefined thixotropic state, which meant that all subsequent measurements would lie on an unknown baseline. In repositioning, we did not expect any thixotropic effects, so the question simply became, was there any difference in outcome between unconditioned and conditioned trials?

#### One-arm repositioning task

Repositioning used the simplest of the three arrangements of the apparatus (Fig. 1C). It involved only one paddle and one arm strapped to it. The measurement and recording of the angular errors were the same as in the other experiments. Similarly, the method of conditioning of the arm was similar. The blindfolded subject's arm was moved to a position of extreme flexion or extension (125°, 5°), the paddle supporting the arm was locked in position by the struts and the participant carried out isometric contractions of elbow flexors and then extensors. Once the arm had relaxed, the paddle was unlocked and moved to a chosen test angle.

Three different conditions were tested. In the first, “none”, no conditioning was used. The arm was simply strapped to the paddle, and moved from its starting position (125° or 5°) by the experimenter to a chosen test angle and the participant asked to remember that angle while the arm was held there for 2 s. The experimenter then returned the arm to its starting position, waited for 2 s and then asked the participant to reproduce the remembered position. For each trial, both the remembered and reproduced positions were recorded.

In the second condition, “after”, learning the location of the test angle was done as before, but once the arm had been returned to the starting position (125° or 5°), both flexors and extensors were conditioned. Two seconds later, the participant was asked to reproduce the remembered position. In this case, the participant moved an arm that had been sensitised in its flexor and extensor muscles so that the afferent signal coming from the arm during the reproduction stage, no longer matched that of the learning stage. In the third condition, “both”, the arm muscles were co-conditioned, both before the memorizing step as well as before the reproduction step. Here, both the learning stage and the reproduction stage were made with an arm whose muscles had been sensitised by conditioning. For each condition and each test angle, the trials were repeated three times. Half the participants used their dominant arm as the test arm, half used the non-dominant arm.

The repositioning experiment was done in two parts. It was decided to avoid the problem encountered at extreme test angles in matching and pointing of having an arm that underwent conditioning contractions but was not moved. In repositioning, when the starting position was 125°, test angles of 95°, 65°, 35° and 5° were used. When the starting position was 5°, test angles of 35°, 65°, 95° and 125° were used. It meant that the arm always underwent at least 30° of movement before reaching the test angle. It did reduce the total number of trials for each participant from 30 to 24.

#### Determination of position error

In the two-arm matching task, once the participant had achieved a satisfactory match, an electronic marker was placed on the recorded position trace. The angle of both reference and indicator forearms (relative to horizontal) were recorded, as well as their difference, that is, reference angle minus indicator angle. Here, the convention was adopted that positive errors were where the indicator had been placed in a more extended position than the reference arm, negative errors were where the indicator had adopted a more flexed position. A similar convention was adopted in pointing; a positive error referred to the indicator paddle being placed at a more extended angle than the true position of the hidden arm. The angles for the reference arm and indicator paddle were noted, as well as their difference; in repositioning, the learned angle was noted, as well as its reproduced value and the difference between the two. Perceived angles or angular differences were plotted against test angle.

#### Statistical analysis and reporting

For each measurement method, a two-way repeated measures ANOVA was used to determine whether there was a significant effect of conditioning or test angle (independent variables) on position matching error (dependent variable). For matching and pointing, conditioning was defined as the conditioned angle (four groups for matching, two groups for pointing), while for repositioning conditioning was the three conditioning types (none, after, before and after). We also tested for an interaction between conditioning and test angle on position error. Where significance was found pairwise comparisons used the Bonferroni adjustment. When additional post hoc tests were required (for significant interactions), group means were compared using paired two-tailed t-tests and the Holm–Bonferroni method to adjust for multiple tests. The significance level applied for all statistical tests was 0.05. Statistical analyses used IBM SPSS version 26.

All values reported in the text or figures are the group mean (± standard deviation) unless otherwise specified.

## Results

### Two-arm matching

#### Effect of muscle conditioning

In the present study, extreme forms of muscle conditioning were used with all three methods of position sense measurement. Under these conditions, the participant was expected to make large position errors. Measurement of position sense with opposite conditioning was compared with a series of control measurements where both arms were conditioned at the same angle, and then both were moved to the same test angle to make the match. It meant that in the controls, the conditioned muscles of each arm were in identical thixotropic states.

During a match, participants were able to maintain the reference arm position reasonably close to the test angle while they moved their indicator to make the match. For example, when the target position of the reference was 125°, the actual angle for the group measured at the time of matching averaged at 125.4° (± 0.8°). We have, therefore, not reported the actual values for the matches, but only position errors between the arms for a given test angle (Fig. 3A).

**Fig. 2:**
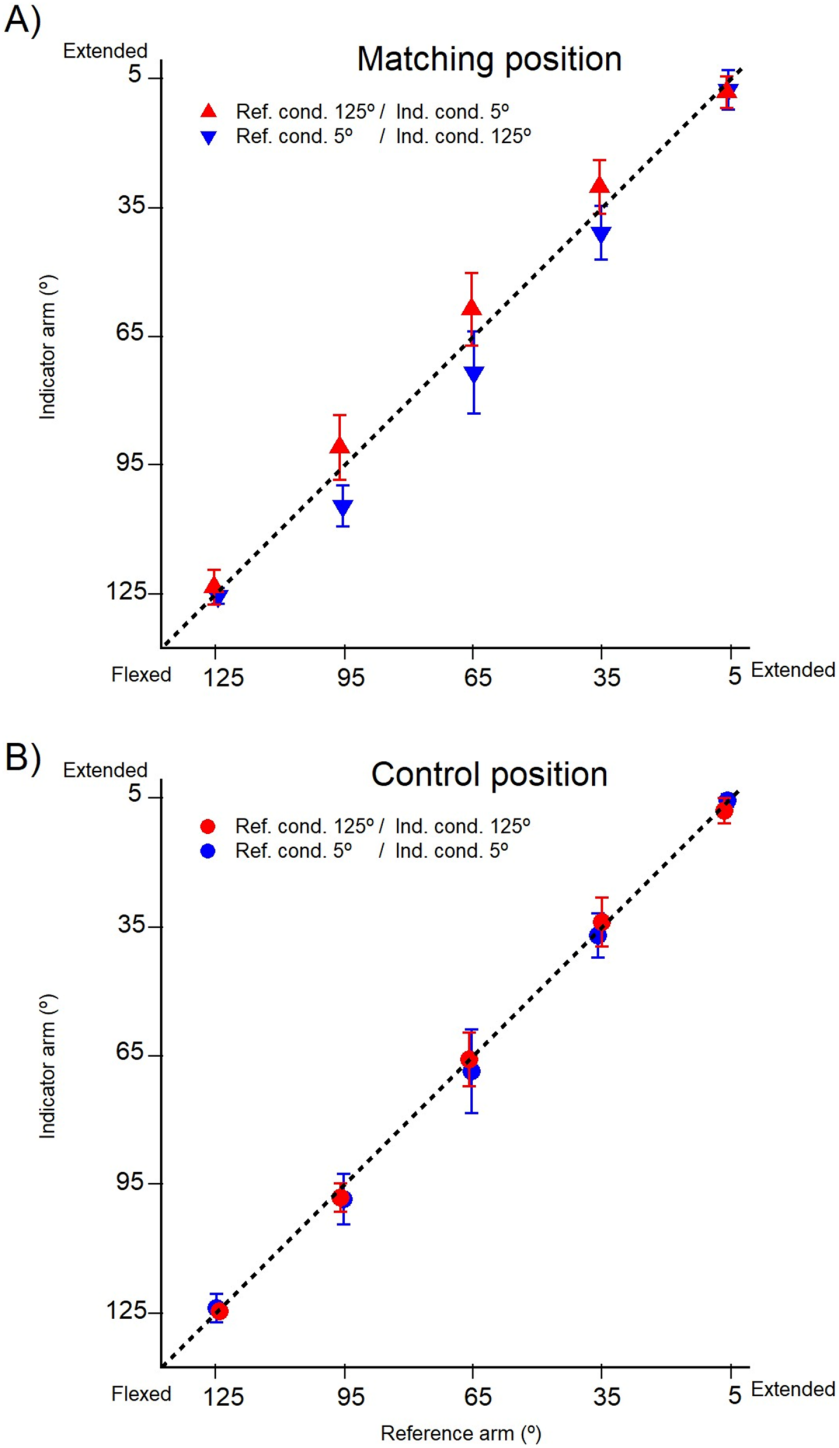
Position sense measured by two-arm matching. **A** Two-arm position matching with arms coming from opposite directions. At the start of a trial, one arm, the reference arm, was conditioned at 125° (flexed) while the other, indicator arm, was conditioned at 5° (extended, red triangles). Conditioning consisted of a half-maximum contraction of elbow flexors and then extensors. After conditioning, the relaxed reference was moved to the test angle and its perceived position was matched by the blindfolded subject’s indicator arm, coming from the opposite direction. Then the conditioning sequence for the two arms was reversed, the reference conditioned at 5°, the indicator at 125° (blue triangles). Test angles, 125°, 95°, 65°, 35°, 5°. Matching positions are shown as means (± SD) for three repeated measurements at a test angle for each of a pool of 11 subjects. Dashed line, line of equality, the position the indicator would have adopted if it had accurately matched the position of the reference. **B** Controls: positions adopted when both arms were conditioned identically. In this experiment both arms were conditioned at 125° (red circles) or at 5° (blue circles), before being moved in the same direction, extension or flexion, to the test angle. It meant that during measurements of limb position, muscles of both arms were in a thixotropically identical state. Test angles as in A. Values shown as means (± SD) for three repetitions pooled for 11 subjects. Dashed line, line of equality

**Fig. 3:**
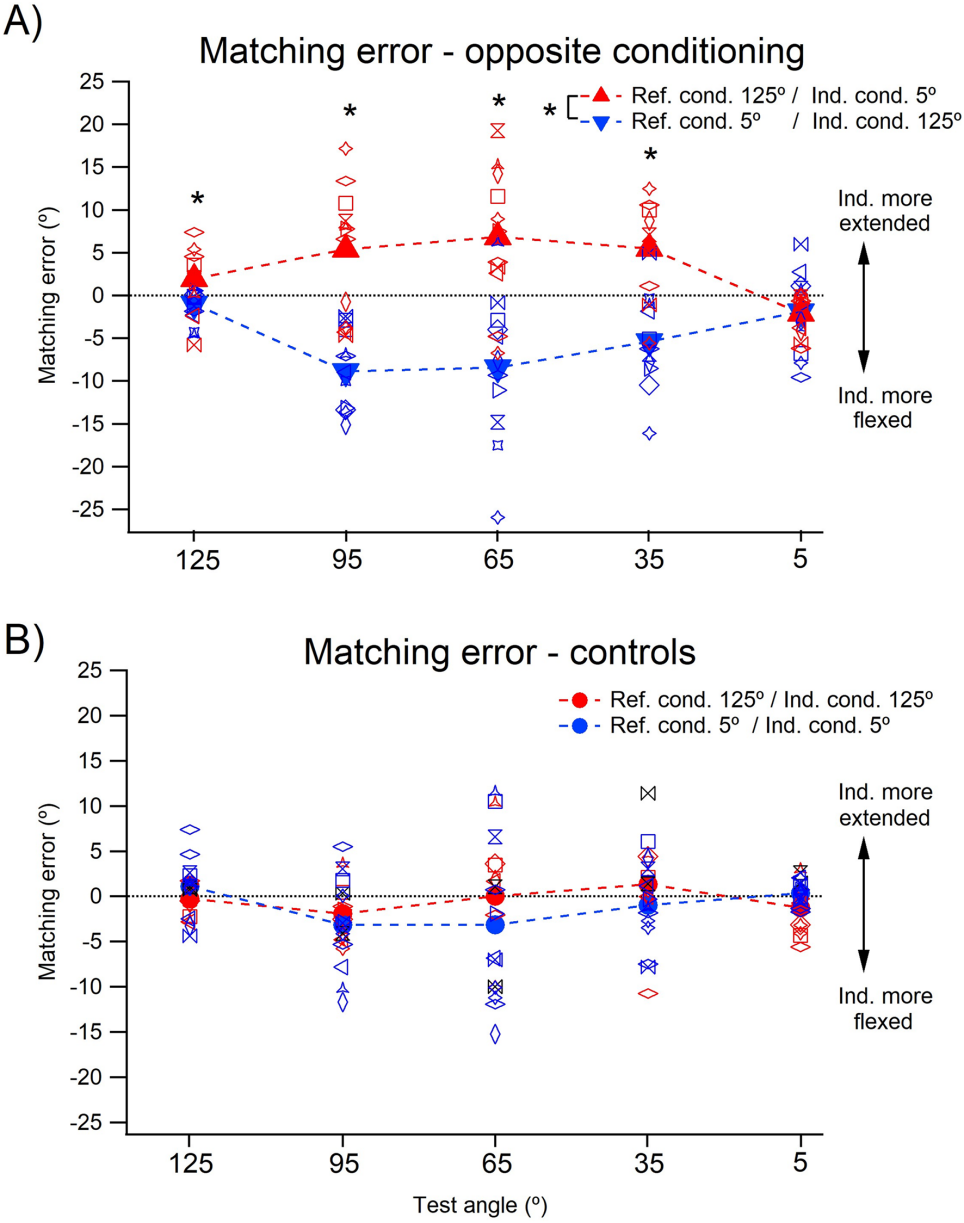
Two-arm matching errors. **A** Matching errors with arms conditioned at opposite ends of the angle range. Here the comparison was not between positions of the two matching arms, but between the errors made in the matches at each of the five test angles. Red symbols, reference arm conditioned at 125° and indicator arm at 5°, blue symbols, reference arm conditioned at 5° and indicator arm at 125°. Values are shown as means for three repeated trials for each of the 11 subjects, as well as group means (filled symbols). Dashed line zero error. Asterisks indicate significant difference between error values for that angle. **B** Controls: matching errors with identically conditioned arms. Both arms were conditioned at either 125° or 5° and both were moved in the same direction to the test angle, to induce in them an identical thixotropic state at the time of matching. Red symbols, arm muscles conditioned at 125°, blue symbols, arm muscles conditioned at 5°. Values shown as means for three repetitions for each of the 11 subjects, as well as the group means (filled symbols). Dashed line, zero error

In the display shown in Fig. 2A, the position of the reference arm is plotted against the indicator arm for 11 participants. The line of equality represents the position the arms would have adopted if they had matched precisely (zero position error). In Fig. 3A are shown the actual errors for a given test angle. The display shows the group means plus the individual means for each of the 11 subjects. In reading the error plots, it should be noted that the display of position error versus forearm position used an expanded scale compared with that in Fig. 2A.

It was found that when the reference arm had been conditioned at 125°, indicator positions lay systematically above the line of proportionality; that is, the participant perceived the position of the reference arm as more extended than it was; when the reference had been conditioned at 5°, it was perceived as more flexed.

#### Effect of muscle conditioning at different test angles

The differences in position errors attributed to muscle conditioning were particularly prominent for the three mid-range angles. When arms were conditioned at the extreme test angles, 125° and 5°, position errors were smaller than in the mid-range. It should be noted that in two of the trials at the extreme angles the reference arm was not moved after co-conditioning (since it was already located at the test angle) and in the other two, the indicator was not moved.

Statistical analysis showed that there was an overall effect of conditioned angle on position error (*F* (3, 30) = 13.95, *p* < 0.001) with no overall effect of test angle on position error (*p* = 0.33). For each of the two forms of opposite conditioning (Fig. 3A), matching errors were significantly different, except for the 5° value. A significant interaction was found between conditioned angle and test angle, (*F* (12, 120) = 6.84, *p* < 0.005). Post hoc tests showed that for the two opposite conditioned groups, matching errors were significantly different for test angles of 125° (*p* = 0.02), 95° (*p* = 0.001), 65° (*p* = 0.001), 35° (*p* = 0.01), but not for 5°.

While there were significant differences in position error attributed to muscle conditioning that varied by test angle, there was no effect of test angle alone (independent of muscle conditioning) on position error.

#### Control trials

In the control measurements, the errors were small (Fig. 2B). Group mean errors and means for each of the 11 subjects are shown for the control matches in Fig. 3B. For the two control groups (Fig. 3B), no significant differences were found between matching errors.

### One-arm pointing

In the pointing tasks, the participant was asked to co-condition their arm hidden behind a screen (Fig. 1B) and the experimenter then moved the relaxed arm to a chosen test angle. With their other hand, the subject rotated a lever moving the pointing paddle to align it with the perceived position of the hidden arm.

#### Effect of muscle conditioning

**Fig. 4:**
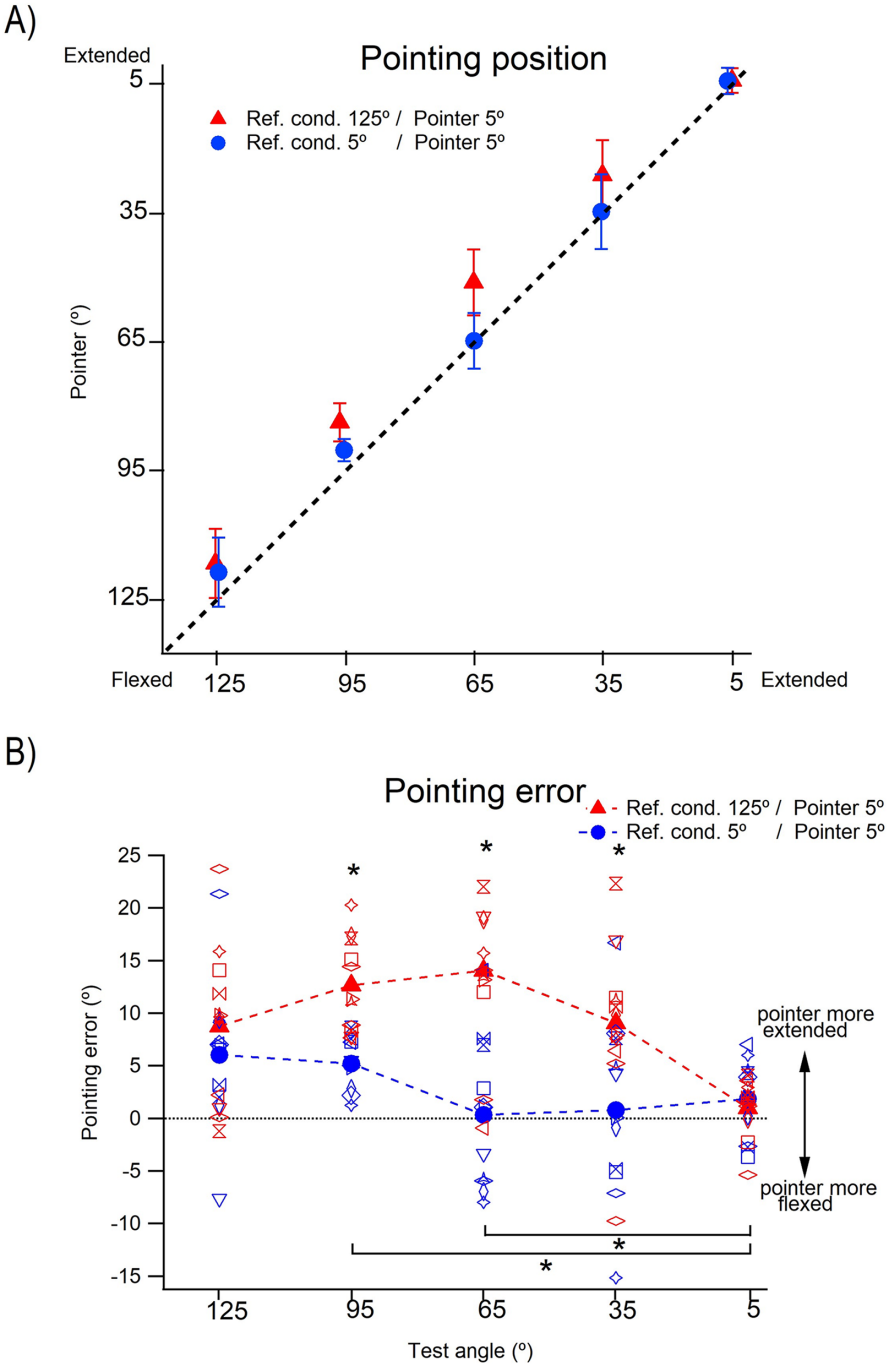
Position sense measured by pointing. **A** Position of a hidden arm after opposite conditioning. The subject indicated with a pointer the perceived position of their arm hidden behind a screen after conditioning of its muscles at the opposite ends of the movement range. Elbow muscles had been co-contracted at 125° and the arm then moved into extension to each of five test angles, 125°, 95°, 65°, 35° and 5° (red triangles), or co-conditioned at 5° and the arm moved into flexion to the test angles (blue circles). Values shown are means (± SD) for three repetitions at each test angle, pooled for 11 subjects. Dashed line, zero error. **B** Pointing errors in arm position after opposite conditioning. Errors made in pointing to the location of the hidden arm at each of the five test angles after it had been conditioned at 125° (red symbols) or at 5° (blue symbols). Values shown as means for three repetitions at a test angle, for each of the 11 subjects, as well as group means (filled symbols). Asterisks indicate significant differences. A further comparison between values after conditioning at 5° and 65° was found to be significant, as was the comparison between the 5° and 95°

Figure 4A shows for the 11 participants, after opposite conditioning, the distribution of pointing errors; these were similar if smaller than for matching. In addition, none of the values for conditioning at 5° lay below the line of equality, in the direction of flexion.

A two-way repeated measures ANOVA showed that there was a significant overall effect of conditioning angle on position errors (*F* (1, 10) = 16.6, *p* = 0.002).

#### Effect of muscle conditioning at different test angles

Average pointing errors are shown in Fig. 4B. Here, as before, both individual means and group means have been shown. A significant interaction was found between conditioned angle and test angle (*F* (4, 40) = 9.80, *p* = 0.0001). Post hoc tests showed that pointing errors for the two forms of conditioning were significantly different at the three intermediate angles, 95° (*p* = 0.001), 65° (*p* = 0.002) and 35° (*p* = 0.02).

#### Effect of test angle

There was an overall effect of test angle on position error (independent of muscle conditioning), with position errors tending to lie above the line of equality (into extension) for all positions. A two-way repeated measures ANOVA showed that there was a significant overall effect of test angle on position errors, (*F* (4, 40 = 3.19, *p* = 0.02). Pairwise comparisons for test angles showed that pointing errors at 5° were significantly different from errors at both 95° and 65° (Fig. 4B). All other comparisons were not significant.

### Repositioning

This method of measuring position sense was rather different from the previous two methods. During the test subjects were not attending an ongoing position signal; they were required to reproduce a remembered forearm position previously presented to them (Fig. 1C). A specific question was whether muscle conditioning carried out before or after the memorising stage would alter recall of that position. Any changes in repositioning errors would imply that the influence of signals from muscle spindles could still be traced to this memory-based task.

#### Effect of muscle conditioning

**Fig. 5:**
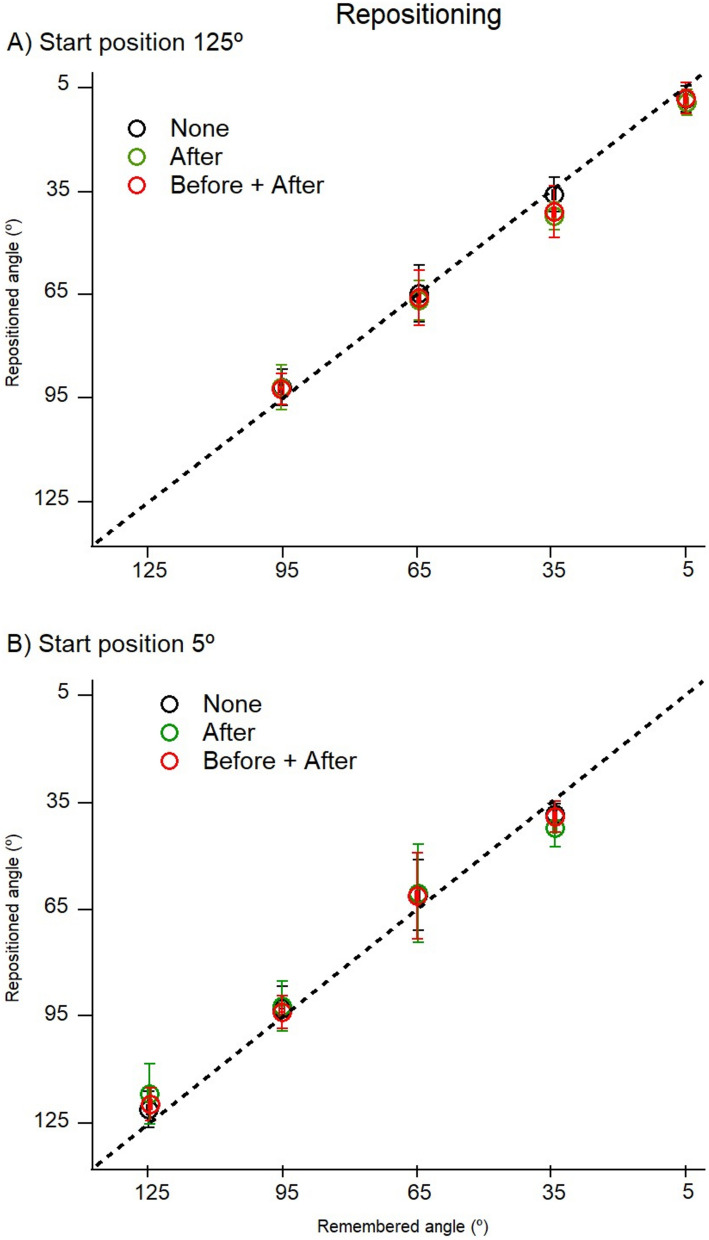
Position sense measured by repositioning. **A** Starting position 125°. Starting at 125°, the forearm was moved in the direction of extension to one of four test angles, 95°, 65°, 35° and 5° and the subject asked to remember that angle. After returning the arm to its starting position they were asked to reproduce the remembered angle. Values shown as means (± SD) for 3 repetitions by each subject, pooled for 11 subjects. Black symbols (‘None’), arm muscles unconditioned, green symbols (‘After’), arm muscles co-conditioned at 125° after the learning stage and before the reproduction stage. red symbols (‘Before + After’), arm muscles conditioned both before the learning stage and before the reproduction stage. Dashed line, zero error. **B** Starting position 5°. The forearm, starting at 5°, was moved in the direction of flexion to each of the four test angles, 35°, 65°, 95° and 125° and the subject asked to remember and reproduce them. Black symbols (‘None’) arm muscles unconditioned, green symbols (‘After’), arm muscles conditioned at 5° after the learning stage, red symbols (‘Before + After’), arm muscles conditioned both before and after the learning stage. Values shown as means (± SD) for 3 repetitions for 11 subjects. Dashed line, line of equality, if the remembered position had been accurately reproduced

The mean repositioning values for 11 participants after conditioning contractions at 125° are shown in Fig. 5A and for 5° are shown in Fig. 5B. The first impression of the distribution of position values was that regardless of start position (125° or 5°), in comparison with matching and pointing values, position errors were relatively small. Secondly, there were no obviously discernible differences in the distribution of errors for “none”, “after” or “both”.

**Fig. 6:**
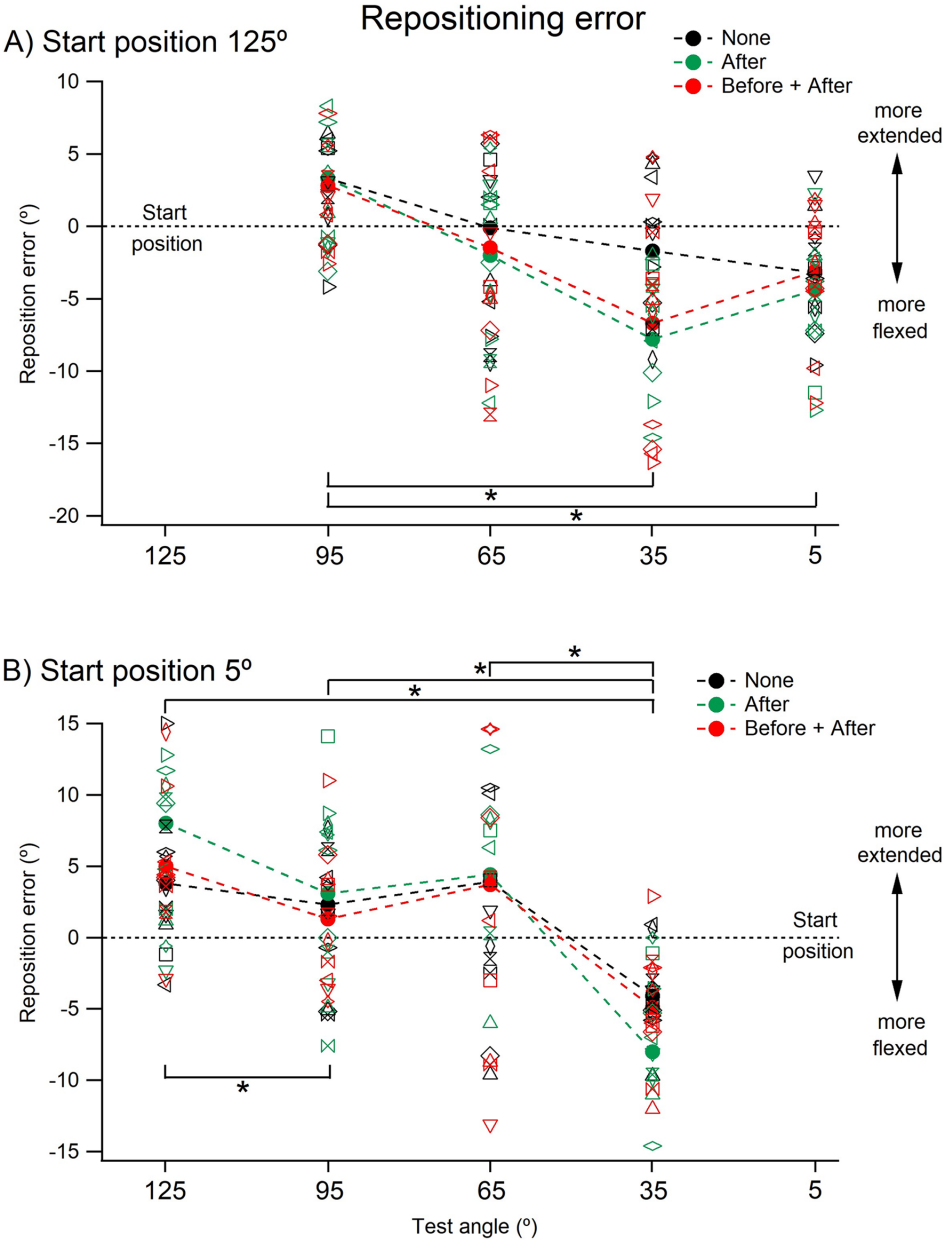
Repositioning errors. **A** Starting position 125°. Repositioning errors made at each of the four test angles, coming from the starting position of 125°, under the three conditions. Individual means for 3 repetitions for each of the 11 subjects are shown together with the group means (filled symbols). Black symbols (‘None’), indicate errors with elbow muscles left unconditioned, green symbols (‘After’), errors where arm muscles were conditioned at 125° before the reproduction stage and red symbols (‘Before + After’) where arm muscles were conditioned at 125° both before and after the learning stage. Because of the overlap between values, for each colour, values have been joined by a dotted line. Horizontal dashed line, zero error. **B** Starting angle 5°. Repositioning errors as in **A**. Starting angle 5°; conditioning for ‘Before’ as well as for ‘Before + After’ was carried out at 5°. Values shown as means for 3 repetitions for 11 subjects, together with group means (filled symbols). Values for each colour joined by dotted lines. Horizontal dashed line, zero error

Displays of the errors for individual subjects as well as for the group are shown in Fig. 6A, B. This revealed differences in values for the three conditions, “none”, “after” and “both” more clearly. Here, again, it should be remembered that the position error plots were displayed on an expanded scale compared with the arm position plots in Fig. 5, and most errors were less than 5°. In A are shown the errors when conditioning was carried out at 125°, in B the conditioning angle was 5°.

#### Effect of muscle conditioning at different test angles

It can be seen in Fig. 6A (start angle 125°) that at test angles of 35° and 65° errors for “none” lay in a more extended position compared with errors at the other test angles.

In Fig. 6B (start angle 5°), there was no obvious difference in the error distributions for “none”, compared with the other two conditions.

A two-way repeated measures ANOVA showed that for a starting angle of 125° (flexed elbow) there was a significant effect both of conditioning type, [“none”, “after” or “both”; F (2, 30) = 3.91, *p* < 0.05)] and of test angle on repositioning errors [*F* (3, 30) = 11.35, *p* < 0.01], with no significant interaction between conditioning type and test angle (*p* = 0.159). Paired comparisons, however, did not show any significant differences between any of the conditioning types. Paired comparisons for remembered angles showed that the position error at 95° was significantly different from that at 35° (*p* < 0.05) and 5° (*p* < 0.05).

For the starting angle of 5°, there was no significant effect of conditioning type on repositioning errors.

## Discussion

The aims of this project were threefold; first, we wanted to examine the influence of thixotropic conditioning on each of three commonly used methods of measuring human position sense at the elbow joint and see whether it influenced position errors. Secondly, we wanted to compare position errors over the full working range of forearm movement, looking for changes in the distribution of errors at different muscle lengths. Thirdly, we wanted to compare the degree of preservation of thixotropic patterns in the position errors observed with each method.

Thixotropic conditioning alters the maintained rates of discharge in passive spindles. Thixotropy-dependent position errors are an expression of those changes in rates (Gregory et al. 1988). A first question was, did position errors generated with all three methods show evidence of thixotropy-related errors, that is, input from spindles? The answer was, “yes!”. This was an important conclusion since we don’t have any other, non-spindle explanation, for the generation of position sense (but see Gandevia et al. 2006). The second question was, “is the size of thixotropy-dependent errors always the same with each method of measurement? Here the answer was, “no!”. Why might that be? Our interpretation is that the measured value of position errors depends on two factors, the input provided by spindle discharges and the central processing of that information. Our conclusion was that with the three methods used here to study position sense, there were significant differences in the amount of central processing the position signal had undergone.

### Muscle conditioning effects on position errors for two-arm matching

This is the first study of position sense in a matching task, measured over the full working range of a joint, covering 120° of forearm movement at the elbow. From inspection of the pooled data in Fig. 3a, it is apparent that for the three mid-range angles, (35°, 65°, 95°), opposite conditioning produced errors of about ± 6°, representing an error range of 12° at each test angle. Furthermore, when the reference arm was flexion conditioned (125°) and the indicator extension conditioned (5°), the matching errors always lay in the direction of forearm extension; after extension conditioning of the reference and flexion conditioning of the indicator, errors lay in the direction of flexion. The systematic changes in the direction of the errors, after the two forms of conditioning, argue in support of thixotropic influences from muscle spindles as responsible for that distribution (Gregory et al. 1988).

Early observations of what turned out to be thixotropic behaviour, referred to “post contraction sensory discharge” (Hutton et al. 1973). This was the increase in spindle discharge following a muscle contraction. We now know that this happens because of the take-up of slack in the muscle and its spindles by the contraction (Proske and Gandevia 2018). Since muscle spindles are stretch receptors signalling muscle length (Matthews 1988), in a position sense experiment, the higher spindle discharge rate after a conditioning contraction is perceived by the subject as a longer muscle; for elbow flexors, this meant a more extended forearm and for elbow extensors a more flexed forearm. It is an illusory effect, similar to that produced by vibration, since after the contraction there was the perception of a displaced forearm, yet the arm had not moved.

The regular occurrence of post-contraction sensory discharge means that in any study of position sense in a passive limb, if limb muscles are left unconditioned, measured values of position sense will be biased towards perception of a shorter, less stretched muscle than is actually the case. The extent to which this happens depends on the immediate history of contraction and length changes of the muscle. This is a frequently overlooked issue in studies of proprioception and it is known that to avoid such effects, standing torque levels of 5–10% of maximum are necessary (Jahnke et al. 1989).

We are proposing that, in the absence of muscle conditioning, the brain makes use of an established spindle discharge rate—muscle length (joint angle) relation. That is, a given forearm position is attributed to a particular, maintained rate of spindle discharge. This relationship is laid down during development, based on a young animal viewing its arm movements and perceiving the accompanying sensations generated by the movements (Held and Bauer 1967). In the adult, at a given test angle, if spindle discharge rates are raised above the expected, calibrated value for that angle by a muscle contraction, this will be misinterpreted by the brain as a longer, more stretched muscle (Banks et al. 2021, Fig. 10).

There is some evidence in support of the existence of a calibrated spindle discharge: muscle length relation. Two independent studies have reported that the increase in spindle discharge evoked by vibration can lead to perception of joint angles beyond the anatomical limit of movement at a joint (Craske 1977; Lackner and DiZio 1992). These observations suggest that as a muscle is stretched to long lengths, there is no information contained within the spindle signal alerting the brain of the approaching limit. When vibration evokes a rate of spindle discharge that implies a length beyond the limit, the discharge rate: muscle length relation operating within the limits of limb movement, is extrapolated by the brain to determine the anatomically impossible value (Craske 1977). Presumably, it is left to joint receptors to signal the approaching limits of movement at a joint (Fuentes and Bastian 2010; Proske 2023).

After co-conditioning of the reference arm in a flexed position (125°), it is moved in the direction of extension to the test angle. Its flexor muscles have been stretched by the extension movement and they are, therefore, generating high levels of spindle activity at the test angle, higher than normal, leading the subject to believe that their arm is more extended than is the case. The indicator arm comes from the opposite direction (5°) to make a match, and this time, it is the extensors which are stretched by the movement, making the subject think their arm is more flexed. At the test angle, therefore, the reference is perceived as overextended by about 3°, the indicator overflexed by 3°. In making the match, the subject stops their indicator arm too early, 6° short of the actual test angle, as a result of influences coming from both arms. The same argument can be applied if the reference is coming from 5° and the indicator from 125°, but the direction of the errors will be reversed and lie 6° in the direction of flexion.

Such reversals of errors have been observed previously. In a study of position sense measured with arm movements in the horizontal plane, with the reference arm flexion conditioned and the indicator extension conditioned, errors of 11.6° into extension were observed (Allen et al. 2007). Reversing conditioning led to 9.5° errors into flexion. Therefore, here, the total error range was 20.1°. In the present study, where position sense was measured in the sagittal plane, when the reference arm was moved from 125° to the test angle, for the three mid-range angles, the mean error was 5.9° into extension; when it was coming from 5°, it was 7.6° into flexion. This gave a total error range of 13.5°. The larger errors in the earlier study are attributed to the fact that position sense was measured in the horizontal plane, in a gravity-neutral posture. In the present experiments, we opted for the more natural situation where the subject had to bear the weight of their forearm themselves. The accompanying muscle activity (5% of maximum, Winter et al. 2005) may have led to some uptake of slack in elbow muscles, thereby reducing the size of thixotropic errors.

When an arm, conditioned at 125°, is moved into extension, its flexors are stretched; at the same time, its extensors will be shortened and, therefore, fall slack, their spindles becoming desensitised. Do the shortened extensors contribute, in any way, to matching errors? Muscle spindles are stretch receptors and they signal muscle lengthening, not shortening (Capaday and Cooke 1981, 1983; Inglis and Frank 1990; Inglis et al. 1991; see also Di Giulio et al. 2009). Therefore, when the arm is moved into extension, flexor spindles will provide the position signal, when it is moved into flexion, extensor spindles will provide the position signal, with no contributions from the slack antagonists.

While matching errors for the three mid-range angles were large, for the two extreme angles, 5° and 125°, errors were small and for 5° insignificant. This was probably because here, in the matching process, one of the arms had not moved, leaving spindles in both of its antagonists sensitised after conditioning. Movement of the other arm to make a match sensitised spindles in one of its muscles and this could be accurately matched with spindles in the stationary arm.

Identical conditioning of the two arms was used as a form of control (Fig. 3b). Here, errors were very much smaller, 2°–3°. This was to be expected since after conditioning, both arms were likely to be in a near identical thixotropic state at each test angle. It is interesting that the small size of the control errors is maintained over the full range of angles tested, including the extreme angles (Fig. 3b). There is no evidence of a change between mid-range and extreme angles. The observation supports the view that for the experiment using opposite conditioning of the two arms (Fig. 3a), a reduction in position errors at the extreme angles (5° and 125°) was, at least in part, attributable to thixotropic effects.

### Muscle conditioning and position errors in a pointing task

First, it is worthwhile to recapitulate what is known about pointing and matching as two separate methods of measuring position sense. We have proposed that indicating the position of one limb by placement of the other in a matching task involves muscle spindles from muscles of both limbs (Proske and Chen 2021). Alignment of the limbs uses the frequency code of the afferents, where an increase in impulse rate is interpreted as a longer muscle and, accordingly, a more extended or flexed joint. It is an accurate mechanism, where the brain determines the degree of alignment of the arms based on differences in afferent signals between them. The mechanism does not appear to involve vision since normally the task is carried out blindfolded and visual distortions presented to the subject before the measurements do not lead to additional matching errors (Velay et al. 1989).

A second means of determining position sense is by pointing to a hidden body part. Most studies claiming a pointing task used, for example, a finger of one hand pointing to the perceived position of the equivalent finger, or other landmarks, on the hidden hand (Longo and Haggard 2010; Ingram et al. 2019). It is thought that vision of the pointing finger is an important contributor to the task. Our own work comes from a background of studies of two-arm matching using alignment of the forearms. For pointing, we wanted to measure the perceived position of the hidden forearm under conditions where there was no opportunity for proprioception in the other arm to be able to make a meaningful contribution. For placement of the pointer paddle, the position information arising from the hidden forearm was presumed to be converted to a visual frame of reference that allowed the subject to align the pointer.

The work of Velay et al. (1989) has shown that the pointing mechanism is susceptible to errors from distortions of the visual field presented immediately before a measurement. Therefore, vision is likely to play a role in the generation of this sense. In addition, there is the suggestion of a memory component in perception of the position of the hidden arm (Velay et al. 1989; Chen et al. 2021). In contrast, there is no evidence that memory plays a role in two-limb matching (Horch et al. 1975; Tsay et al. 2014).

We have previously reported two features of the position signal in pointing: it appeared to be insensitive to thixotropic conditioning of muscles and, for the forearm, pointing errors lay consistently in the direction of arm extension (Tsay et al. 2016; Chen et al. 2021). In the present study, examination of the pooled data for 11 subjects who carried out the pointing task (Fig. 4) shows that at the intermediate test angles there were differences in errors, depending on whether the arm had been conditioned at 125° or at 5°, errors which reached significance at 35°, 65° and 95°. These findings are different from previous observations on pointing (Tsay et al. 2016). In Tsay et al., measurements were made at a single test angle (40°–50°) and the arm had been conditioned at either 90° or 0°, giving a conditioning: test angle range of approximately 45°. The smaller movement range to the test angle may have contributed to the lack of significance in the distribution of position errors in our earlier study. In addition, in the present study, making the comparison between positions of the arm conditioned at opposite ends of its movement range was likely to maximise thixotropic effects. We assume that this was responsible for bringing out thixotropy-dependent errors that we had not seen previously.

In pointing, when the arm was conditioned at 125°, values tended to lie further in the direction of extension compared with after conditioning at 5°. This was a similar pattern to that seen with two-arm matching. The average pointing error into extension for the three mid-range test angles after conditioning at 125° was + 11.9°, which was larger than in the matching study (+ 5.9°). When the arm was conditioned at 5°, the average pointing error was + 2.1°. This was quite different from that for matching where the error was − 7.6°. Therefore, in the mid-range of test angles, there were differences in the ranges of the errors: 13.5° for matching and 9.8° for pointing. It meant that the outcome of the pointing experiment in the present study showed some elements of our previous observations; in pointing differences in errors between the two opposite forms of conditioning tended to be smaller. Perhaps, this was due to the fact that proprioceptive signals from only one arm were involved during pointing. This is supported by the finding that errors attributed to conditioning were similar between matching and pointing if we compare trials where only the reference arm conditioning was manipulated (Fig. 3 vs. Fig. 4b, red triangles and blue circles). In addition, pointing values lay either on the line of equality or above it, in the direction of extension, with no values lying in the direction of flexion, as had been seen in matching (Fig. 3a).

Our earlier studies of pointing suggested that in the mid-range of elbow angles, error values lay superimposed on an offset, in the direction of extension, of between 4° and 10° (Tsay et al. 2016; Chen et al. 2021). We suggest that a similar offset is present in the current pointing data and it is this which accounts for the absence of position errors below zero. The assumption implicit in such an interpretation is that the offset always lies in the direction of extension regardless of the direction of the thixotropic errors.

We do not know why such an offset is present, but it may relate to the volume of spindle afferent traffic generated in forearm antagonist muscles. A higher level of flexor activity would bias the perceived position of the forearm in the direction of extension. In the only available count of spindle numbers in human elbow muscles, flexors contained 20% more spindles than extensors (Voss 1971). Observations supporting the existence of a flexor-biased afferent signal at the forearm come from the illusory responses to vibration; vibrating elbow flexors produced illusions into extension several times larger than illusions into flexion during vibration of elbow extensors (Craske 1977; Lackner and DiZio 1992). A similar offset in perceived arm position could potentially be present in two-arm matching, but since what is measured is the difference in position of the two arms (Proske and Chen 2021), any offset in perceived position would be subtracted out in the matching process.

To summarise, while in pointing the distribution of errors showed evidence of conditioning dependent effects, differences in errors for the two forms of conditioning were smaller and errors lay further in the direction of extension when compared to matching. Here, it must be kept in mind that pointing involved afferent signals from only one arm, while matching involved both arms.

### Muscle conditioning effects on position errors in a repositioning task

The main objective of the present study of position sense using the method of repositioning was to try to determine whether thixotropy played a role at all. At the outset, we had assumed that determining position sense by repositioning, where the subject was asked to remember a given test angle, involved a large memory component. If so, it suggested that the position signal we were dealing with was likely to be a more processed one than for matching or pointing. We, therefore, hypothesised that in repositioning, if there was an influence of thixotropy on position errors, it was likely to be smaller than in matching or pointing. That prediction was fulfilled.

Plots of repositioned against remembered angles (Fig. 5a, b) showed that all values of remembered angles lay close to the line of equality, no matter whether the arm was coming from the direction of flexion or extension. Furthermore, differences in values between the three conditions were small. A little more information was provided by the display of errors (Fig. 6a, b).

When the arm was coming from 125° (Fig. 6a), the error values for “none” were equal to or lay above those for “after” and “both”. Presumably for “none” a memory of forearm position was laid down, based on the perceived level of afferent activity at the test angle coming from the stretched, unconditioned, elbow flexor muscles. For the condition “after”, at the remembered angle, the level of afferent activity would have been higher because of the conditioning contraction (post contraction sensory discharge). Therefore, with the intention of reproducing the muscle length corresponding to the remembered, lower spindle discharge rate, for “after” the subject repositioned the arm at a more flexed angle, where flexor discharges were lower, leading to errors in the direction of flexion compared with “none”. Essentially, the same argument applies to “both”. Here, the subject had already incorporated into their memory the higher level of afferent activity from the conditioning contraction. Therefore, the repositioning error was similar to that for “after” and it remained different from “none”.

If the arm was coming from 5° (Fig. 6b), the muscles undergoing stretch during the movement to the test angle were the extensors. Here, the errors for “after”, would be expected to lie in the direction of extension compared with “none”, since the higher extensor activity after conditioning would indicate a more flexed angle. There was a hint of this at the most flexed angle, but the effect was weak.

There remain unexplained aspects of the error distributions in repositioning. For both the 125° and 5° starting positions, for the more flexed test angles, error values for “none”, “after” and “both”, all tended to lie in the direction of extension and they reversed in the mid-range to lie in the direction of flexion for the more extended angles. Since errors for all three conditions did this, it was unlikely that an explanation involved thixotropy.

To conclude, while evidence for thixotropic effects on position errors in the repositioning task was weak, certainly weaker than in matching or pointing, statistical analysis supported the presence of some influence of thixotropy on the errors, particularly when the starting angle was 125°. This raises the possibility that after conditioning at 125° the influence on repositioning errors from stretched flexors was greater than that from stretched extensors after conditioning at 5° (see above).

There is some evidence in the literature for an influence of muscle spindle signals on repositioning errors (Larish et al. 1984). In a forearm repositioning task in the horizontal plane, repositioning errors were larger if, during the interval between remembering and reproducing the test angle, elbow flexors were vibrated. It was concluded that vibration, a stimulus known to be selective for the primary endings of spindles, was able to interfere with the repositioning mechanism.

### Wider considerations

In everyday life do we ever match the positions of our two arms? Whenever we work with both hands, we bring them together as we manipulate objects and work with tools. To be able to align the arms accurately, bringing the hands to face each other, we are likely to make use of the matching mechanism. However, we do not consciously align our arms to know where they are. If asked, without looking, we always know where each arm is separately.

In their study, Chen et al. (2021) carried out a pointing experiment where the subject indicated the position of the arm hidden behind a screen, not by pointing with the other arm, but by verbally reporting which of a series of lines drawn on the screen lay closest to the perceived position of the arm. The resultant distribution of the errors was similar to that from a standard pointing task.

Such a result leads to two conclusions; one, that in the pointing experiment, signals coming from the arm doing the pointing are not involved in locating position of the hidden arm. Secondly, if we cannot see our arm we just have to think about where it is and we know its position with reasonable accuracy. Here, presumably, at each test angle, the afferent signals coming from forearm muscles are converted in central sensory areas into muscle length: joint angle information. This information would then be forwarded to a central map indicating arm position relative to the rest of the body, as well as contributing to the sense of body ownership (Butler et al. 2017). In addition, the proprioceptive information has to be converted to a visual frame of reference to allow the subject to identify the appropriate line or move the pointer paddle to the perceived angle. All of this suggests that during a standard two-arm matching task position information from more than one source may become available at the same time; combined signals coming from the two arms during the matching, as well as information about each arm separately, as indicated in pointing.

In considering the different methods of measurement of proprioception, recently a broader view has been taken by Heroux et al. (2022). They proposed that proprioceptive assessments should be considered as low-level or high-level judgements, low-level with a single frame of reference and high-level with multiple frames of reference. According to this scheme, two-arm matching would be a low-level task involving a single, direct comparison between signals coming from the two arms. One-arm pointing would be a high-level task where the centrally recorded information from the muscles of one arm is converted to a visual frame of reference, to allow subjects to place their pointer. Repositioning would also be a high-level task: the muscle length: joint angle information is acquired and stored in memory. The remembered information has to be recalled and compared with that generated during repositioning. Therefore, we have tested three methods of measurement, each with a different level of judgement. The sizes and distribution of the observed thixotropic errors approximately follows this classification.

The present study has pointed out that if the aim of a method of measurement of position sense is to try to draw inferences about the central processing of the afferent signals, it will be important to state which method has been used. What might be the meaning of differences in expression of thixotropic errors in measurements of position sense? It seems that the central conversion of spindle impulses into position sensations can be more or less direct; direct in two-arm matching and less direct in pointing and repositioning. It presumably means that as transmission of the afferent signals progresses centrally, the position information it contains can be accessed at different points, dependent on the requirements of the method. This must be kept in mind when drawing any conclusions.

## Data Availability

The datasets generated during and/or analysed during the current study are available from the corresponding author on reasonable request.
